# Scoping Review of Restorative Justice in Academics and Medicine: A Powerful Tool for Justice Equity Diversity and Inclusion

**DOI:** 10.1089/heq.2023.0071

**Published:** 2023-09-29

**Authors:** Gregory Sawin, Christopher L. Klasson, Samantha Kaplan, Jennifer Larson Sawin, Ann Brown, Sonoo Thadaney Israni, Jessica Schonberg, Ada Gregory

**Affiliations:** ^1^ Department of Family Medicine and Community Health, Duke University School of Medicine, Durham, North Carolina, USA.; ^2^ University of Iowa, Carver College of Medicine, Iowa City, Iowa, USA.; ^3^ Duke University Medical Center Library and Archives, Duke University School of Medicine, Durham, North Carolina, USA.; ^4^ Independent Researcher and Restorative Justice Consultant, Durham, North Carolina, USA.; ^5^ Department of Medicine, Duke University School of Medicine, Durham, North Carolina, USA.; ^6^ Stanford University School of Medicine, Stanford, California, USA.; ^7^ Rx for RJ Initiative, University of San Diego, San Diego, California, USA.; ^8^ Office for Faculty, Duke University School of Medicine, Durham, North Carolina, USA.; ^9^ Kenan Institute for Ethics, Duke University, Durham, North Carolina, USA.

**Keywords:** restorative justice, professionalism, JEDI (justice, equity, diversity, inclusion), harm, malpractice

## Abstract

**Purpose:**

Restorative Justice (RJ) as a practice and mindset is growing within academic medicine and health care. The authors aim to categorize the extent to which RJ training and practices have been researched, explored, and applied within health care, medicine, and academic contexts.

**Methods:**

In July 2021, the authors conducted a scoping literature review, searching four databases for peer-reviewed articles and book chapters discussing RJ. Authors also used bibliography searches and personal knowledge to add relevant work. Reviewers independently screened article titles and abstracts, assessing the full texts of potentially eligible articles with inclusion and exclusion criteria. From each included article, authors extracted the publication year, first author's country of origin, specific screening criteria met, and the depth with which it discussed RJ.

**Results:**

From 599 articles screened, 39 articles, and books were included (published 2001–2021). Twenty-five (64%) articles discussed RJ theory with few describing application practices with substantial depth. Ten (26%) articles only referenced the term “restorative justice” and seven (18%) discussed legal applications in health care. Fifty-four percent were from outside the United States. Articles tended to describe RJ uses to address harm and often missed the opportunity to explore RJ's capacity to proactively build community and culture that helps prevent harm.

**Conclusions:**

RJ in health care is a rapidly expanding field that offers a framework capable of building stronger communities, authentically preventing and responding to harm, inviting radical inclusion of diverse participants to build shared understanding and culture, and ameliorate some of the most toxic and unproductive hierarchical practices in academics and medicine. Most literature calls to RJ for help to respond to harm, although there are very few well-designed and evaluated implementations. Investment in RJ practices holds significant promise to steer our historically hierarchical, “othering” and imperfect systems to align with values of justice (vs. punishment), equity, diversity, and inclusion.

## Introduction: A Primer on Restorative Justice Origins, Principles, Practices, and Effectiveness

Anticipating not all readers may be familiar with restorative justice (RJ), this section starts with fundamentals. Howard Zehr, widely considered to be the “grandfather” of RJ in modern day United States, has suggested that RJ is not a *means* to a certain outcome (i.e., a map with directions to achieve reduced recidivism in a criminal justice context), but rather a *framework* that produces those outcomes by promoting desirable shared values (i.e., a compass that points us toward mutual concern for one another).

This article offers a brief primer on RJ principles and practices, surveys the literature on RJ appearing as a term in academic medicine and health care contexts, and concludes with a discussion of how RJ could be used in a health care context to build a new mindset of JEDI (justice, equity, diversity and inclusion) when navigating culture and community, and to address harms and inequities. In this section, the authors examine RJ's principles, origins, practices, and effectiveness.^1–5^

### RJ principles

While definitions of RJ vary, most explanations share widely accepted principles. Chiefly, RJ relies on a view of the community as a web of relationships and as such, the actions of one individual—whether harmful or uplifting—can often affect that entire web of relationships. Harm is primarily a violation of shared values between people; it is not just a breach of a community's rule (which we usually call “wrongdoing” requiring discipline) or a society's code of law (which we usually call “crime” requiring a criminal justice proceeding). With an eye to rebuilding community relationships in the wake of harm, RJ seeks to include those most affected in the community: those harmed, those responsible for the harm, and other stakeholders like family members, neighbors, coworkers, teachers, mentors, and community leaders. Howard Zehr describes RJ as a set of three pillars:^1^

(1) Harms and Needs: RJ seeks to acknowledge harms and resulting needs of people involved in an event. The guiding questions are the following: what occurred, who was harmed, and what do they need?
(2) Obligations: In addressing harm, RJ then focuses on obligations among stakeholders (affected and responsible parties). The guiding question here is now that we understand the harm and needs, who is obliged to meet those needs in an attempt to restore relationships? The offender's obligation to the victim is primary, but RJ principles also explore the communal, institutional, and societal obligations that may have *set up* the conditions for the harm to occur (i.e., moving beyond “bad apple” explanations that risk scapegoating an individual, to explore variables that may have set the stage for the offense), while also ensuring proper resources, reflection and support are present for the responsible party to reasonably meet all obligations.
(3) Engagement: RJ principles suggest that those who are affected by a harm should be meaningfully engaged in seeking to redress the harm. The guiding question here is the following: who was affected by harm, and how do they wish to be engaged in seeking justice and healing? While some practices include direct face-to-face dialogue, levels of engagement may be quite varied and creative (e.g., an exchange of letters, video conferencing, the use of surrogate representatives, etc.).

### RJ origins

The origins of RJ lie firmly with indigenous cultures around the world. Many expressions of RJ principles are especially indebted to the First Nations in Canada and the northern United States,^1^ the Zulu, Xhosa, Tswana, and Venda in Africa,^3^ and the Maori of New Zealand;^1^ these peoples have shaped the array of current RJ practices such as the “circle process” format, the use of a talking piece as a tool to guide inclusive and constructive conversation, and the inclusion of community members at large for support. New Zealand's current legal system is anchored in RJ.^6^

The United States and Canada began incorporating formal RJ measures in the criminal justice system in the 1980s.^7,8^ RJ practices contrast traditional retributive systems widely practiced in the United States and other western nations. Applications of RJ principles have expanded exponentially in the last few decades, in particular, with growing use in K-12 and higher education systems, social work, social justice, workplaces, and even architecture.^9^ This expansion occurred organically and with minimal, if any, funding, organizational prioritization, leadership support or national-level coordination.

This growth shows RJ's value: its adaptability to cultural context, customizability to case-specific circumstances, and shared ownership by all relevant stakeholders. RJ practices have begun to seep into the field of medicine, perhaps best exemplified by the recent Restorative Justice in Academic Medicine (RJAM) Collaborative, organized by the Association of American Medical Colleges (AAMC) in partnership with University of San Diego's “Rx for RJ” initiative.^10^

### RJ practices

RJ practices (or “restorative practices”) are the techniques and processes that emerge from the framework of RJ principles. Kay Pranis articulates that these practices are the mechanisms through which RJ promotes people acting in accordance with their ideals and values,^4^ actions which facilitate community building and conflict resolution. Restorative practices can be scaled and variably applied with degrees of structure and formality to meet the needs of the community as well as the severity of the harm. Moreover, RJ practices are not limited to *responding* to harm. Rather, they can be used to *proactively* build community, working through perceived differences among people, using shared decision-making anchored in shared values and principles, and discussing difficult issues.

In this sense, the practices are not limited to responding to any singular “harm” but can address broadly felt harms or experiences by sharing perspectives grounded in lived experiences or storytelling. By using RJ to intentionally build community, groups can proactively grow a culture that has the potential to reduce inequities, prevent harm, and create more welcoming environments.

**FIG. 1.**
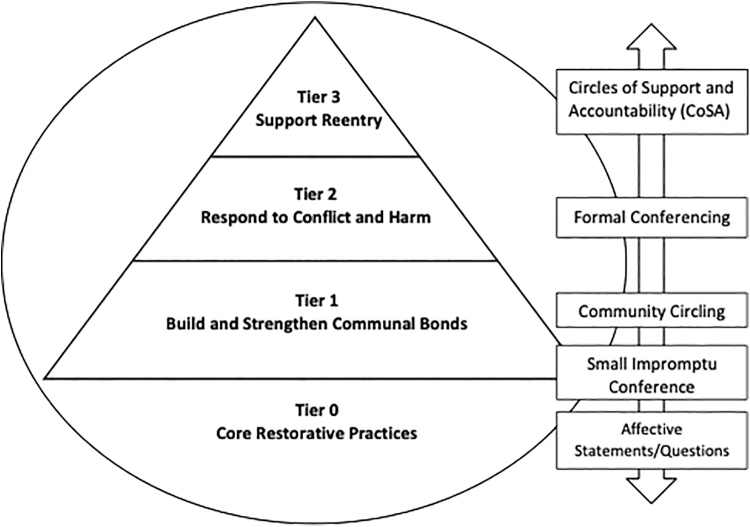
Amended RJ practices pyramid shows a continuum of practices to apply in academic contexts with increasing degrees of formality and structure. Note that these practices are complementary and build from each other, as illustrated by the *double-sided arrow*. These four tiers of restorative practices are the lens through which data are analyzed later in this article. RJ, restorative justice.

Concerning applications in an academic medicine context, David Acosta and David Karp propose a continuum of restorative practices, arranged as a tiered pyramid, where the aim is to build relationships, respond to harm and misconduct, and offer structured reentry for those who have caused harm in a community.^11^ Authors for this article suggest amending the pyramid to make the implicit more explicit by including a foundational tier, which is really more a sphere, of Core Restorative Practices or an RJ Mindset (Fig. 1) that supports and infuses its proactive applications at all tiers. The remaining tiers build upon Karp and Acosta's pyramid with each tier representing increasing degrees of formality, structure, necessary training of convenors, and preparation of the stakeholders.

At the pyramid's base, Tier 1 practices (e.g., circle processes) build community and mutual understanding. Tier 2 practices (e.g., restorative conferencing) are designed to address incidents of harm and wrongdoing. Finally, Tier 3 practices assist reentry into a community by an individual who has caused harm.

Many academic centers seek RJ to address new conflicts, looking to start with Tier 2. In Tier 2 approaches, affective statements and affective questions are the building blocks of high-quality restorative communication (which shares many commonalities with the methods of nonviolent communication).^12^ Sometimes called “I”-statements (e.g., “I feel …” or “I need …”), such language centers on one's own perspective, lived experience, and feelings that grow from met or unmet expectations, affirmed, or challenged values and needs. Restorative processes often open with an appeal to individual participants to communicate using affective statements, to avoid generalities, and to steer clear of attributing experience, thought, or words to someone else.

Similarly, affective questions are open-ended invitations that promote individuals sharing their own perspective and needs (e.g., “Help me understand more about … .” Or, “What is most important to you around this issue?” Or, “What do you need right now?”). Such questions can move a conversation from seemingly intransigent positions that reveal little about the stakeholders, to exploring core human needs that can be shared, heard, and met. Similar to the concept of Appreciative Inquiry,^13^ affective questions and statements can be transformative and useful at a family dinner table, in a boardroom, the principal's office, the “C-Suite,” and in medicine and higher education.

Throughout the “pyramid” of restorative practices, certain core values are consistent. Per Pranis, the “values of respect, maintaining individual dignity, inclusion, and nondomination [all] create a space in which participants are more likely to access the best within themselves.”^4^ These values undergird practices that fundamentally change how people behave, interact with one another, foster authentic relationships, build community, and respond to harm.

### RJ “effectiveness”

A word about RJ “effectiveness” seems important at this stage. Along with the field itself, RJ literature is rapidly expanding and evolving. For better or for worse, much of RJ literature has shifted from a discussion of core concepts and principles (i.e., answering the question, “What is RJ?”) toward a focus on practices that produce measurable outcomes as an indicator of effectiveness (i.e., answering the question, “Does RJ work?”). In the medical arena, this progression of discourse from “discovery” to “outcome” parallels early-stage trials that first explore the safety and effects of a new drug, with a later shift in focus to individual and ultimately population health outcomes. Beyond the medical context, there is a trend in other social sciences toward government agencies lauding, tracking, and funding evidence-based practices and programs that meet rigorous social science standards and demonstrate desirable outcomes.^14^

Of course, while the field of RJ should continue to examine “effectiveness” and outcomes (e.g., research to show reduced recidivism,^15,16^ fewer school expulsions,^17^ high “satisfaction” rates among involved victims, or high percentage of agreements fulfilled), an *exclusive* focus on RJ's quantitative outcomes is myopic, and limits RJ's potential to tap into community wisdom and knowledge to bring about the kind of transformative impact and culture change that is needed.^18^

Although trickier to quantify, authors strongly encourage proactive and robust mixed methods evaluation of RJ programs. Within an academic or health care context, measures of success should be tailored to assess impact on organizational culture, with a specific focus on whether RJ can contribute to change that helps organizations embody their stated values. Beyond evaluating specific training, broader evidence can be gleaned from “culture-pulse” surveys, qualitative data collection, and tracking reporting of microaggressions, complaints, or engagement with ombudspeople.

We must also be mindful that many of our normal operating procedures often go unevaluated, so a strident insistence on the field of RJ documenting its successful quantitative outcomes sets a double standard against “usual practice” that is often itself lacking in evidence and evaluation, or worse, is perpetuated as the norm despite evidence of harm. Within health professions,, this concept of unevaluated efforts applies strongly within diversity, equity, and inclusion efforts as recent work has illustrated the ever-present burden of microaggressions, lack of belonging, and other barriers that minoritized individuals face in academic medicine.^19,20^ Moreover, physician wellness and burnout approaches proved insufficient as burnout is increasingly impacting all health care workers.^21–23^ The authors of this article argue that RJ's practices and values provide a useful framework for bolstering existing efforts in these areas.

## Materials and Methods: Surveying the Literature

The study team used a scoping review methodology because of its strength in finding themes and broad insights in the literature for emerging topics, such as RJ in academic medicine and health care. The study team completed the review adhering to the practices described by Arksey and O'Malley,^24^ and Levac et al.^25^ in their advancement of that framework. The five steps of that process are outlined below. Findings were reported according to Preferred Reporting Items for Systematic reviews and Meta-Analyses (PRISMA) reporting guidelines for scoping reviews.^26^

### Research question

The project's guiding research question was “How is restorative justice being explored and applied in academic medicine and healthcare settings, and what themes appear in the literature?”

### Identifying relevant studies

A medical librarian with expertise in systematic searching crafted a strategy using the term “restorative justice” and relevant subject headings when available. For the larger, interdisciplinary database Scopus, an additional search string pertaining to health was included to refine the results. The librarian searched MEDLINE via PubMed, Embase via Elsevier, APA PsycINFO via EBSCO, and Scopus via Elsevier from inception to July 7, 2021. All results were compiled in EndNote and imported into Covidence. CINAHL was not included as the study aimed to focus on hospitals and environments related to physician practice and training—although studies pertaining to nursing or other clinical environments were not excluded. For a supplementary search strategy, researchers identified additional articles via an initial Google Scholar search in June 2021,^27^ articles and a book that study team members were previously aware of,^11,28–32^ and bibliographic searches of included articles.^33,34^

### Study selection

The screening included abstracts and articles that address the use of RJ, restorative practices, and restorative frameworks within academic medicine, medical practice, public health, or general academia. Note that public health only met inclusion criteria when dealing directly with health care systems, not public health impacts of RJ application within criminal justice, educational systems, or other systems. General academia literature (i.e., meaning university-level institutions) were included to capture RJ's presence in academic environments.

Excluded articles were those that did not mention or describe RJ, and those that contextualized RJ outside of the defined settings (e.g., K-12 education, or criminal justice). There were no inclusion or exclusion criteria for methodological and analytical quality or approach.

The screening process involved two research team members reading abstracts, and then voting as individuals on whether to include or exclude articles. Conflicts in voting were discussed between the reviewers to reach a consensus. The articles that passed abstract screening were then read in full and once again voted on. Articles that were only an abstract (e.g., a published abstract from an oral presentation at a conference) or editorial were included, and articles that had a non-English full text available were excluded.

### Data collection

From the included studies, a data extraction form was created using Excel Software (Version 16.56, Redmond, WA). The following data were collected: Year, Primary Author's Country of Origin, Screening Criteria, Levels of RJ (as described in Fig. 1), and Described Purpose. Any discrepancies in data interpretation were discussed and resolved.

## Results

**FIG. 2.**
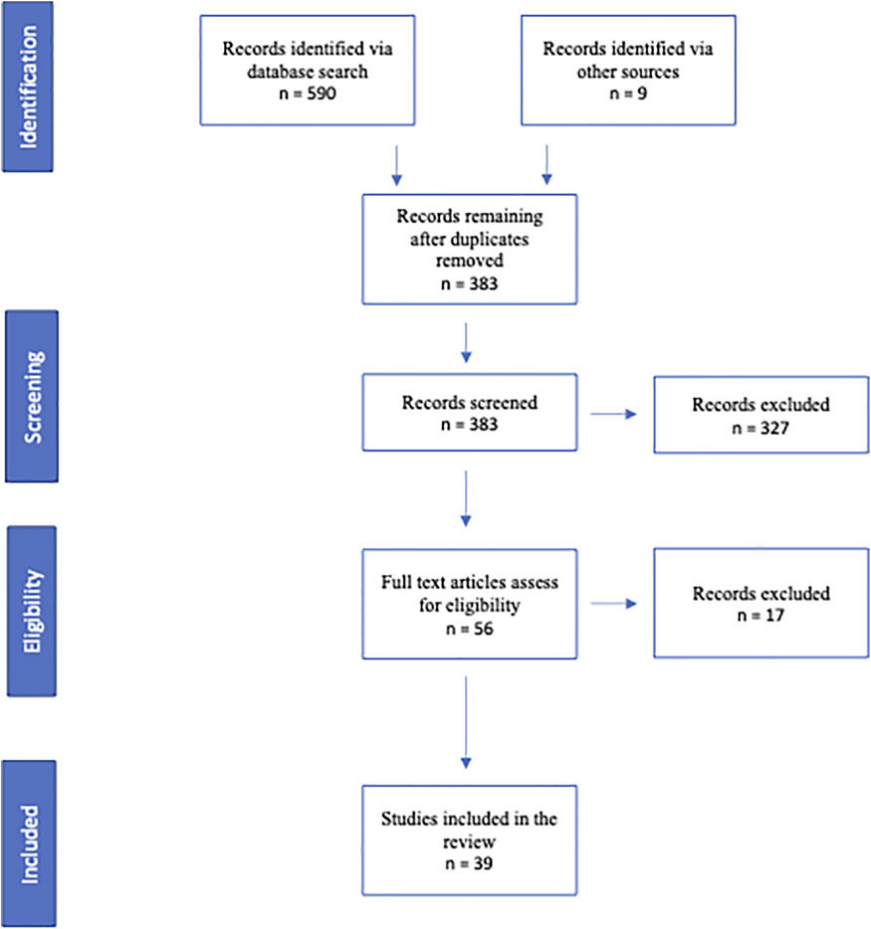
PRISMA flowchart summarizing scoping review process. PRISMA, Preferred Reporting Items for Systematic reviews and Meta-Analyses.

The original search in July 2021 yielded 383 citations. After screening the titles and abstracts, 52 citations remained, based on inclusion and exclusion criteria. Out of those 52, 3 articles were excluded because the full text was not available in English, 13 were excluded because they did not discuss RJ in the context of interest, and one was excluded because, despite having the right setting, the outcome focus was outside of the scope of interest. Thus, 39 articles were included in the review (Fig. 2).

### Article characteristics

Of the 39 included articles, the first authors were from 9 different countries: United States (*N*=18, 46% of articles), Australia (6, 15%), the United Kingdom (5, 13%), Canada (5, 13%), Indonesia (1, 3%), Ireland (1, 3%), Italy (1, 3%), New Zealand (1, 3%), and South Africa (1, 3%). Publication years of the included articles ranged from 2001 to 2021 with 82% of the articles published after 2011. Included articles covered five topics: Medicine (21, 54%), Academic Medicine (6, 15%), General Academia (5, 13%), Public Health (4, 10%), and Research (3, 8%).

### Depth and nature of RJ discussion

Included articles were analyzed for the level they engaged with RJ theory and practices using the Tiers portrayed in Figure 1 and were also classified based on categories of RJ theory, legal context, or broad mention of RJ. Concerning Figure 1, 3 (8%) of the articles discussed Tier 0 (Core Restorative Practices), 9 (23%) discussed Tier 1 (Build and Strengthen Communal Bonds), 14 (36%) discussed Tier 2 (Respond to Conflict and Harm), and 7 (18%) discussed Tier 3 (Support Reentry). In terms of broader classifications, 10 (26%) of the articles made broad or tangential mentions of RJ, 25 (64%) discussed the theoretical ideas of RJ, and 7 (18%) dealt with legal processes or implications (Table 1).

**Table 1.** Article Details and Assessment of Restorative Justice Depth

Title	Year	First author country of origin	Screening criteria	Tiers(s)	Article description
The Little Book of Restorative Justice for Colleges and Universities, Second Edition: Repairing Harm and Rebuilding Trust in Response to Student Misconduct^35^	2019	United States	General Academia	Theory, 0, 1, 2, 3	Book: practical guide to implementing RJ practices and programs in colleges and universities to respond to student misconduct. Includes descriptions of three models (conferencing, circles and boards), instructions for facilitation as well as approach to taking a whole-campus approach.
Hearing and Responding to the Stories of Survivors of Surgical Mesh^32^	2019	New Zealand	Medicine	Theory, 1, 2, 3	Ministry of Health used RJ process to explore their responsibilities to patients with complications from surgical mesh. Thirty-two Listening Circles (*n*=246), eight individual meetings (*n*=21) and an online story database (*n*=484) resulting in public sharing of report, development of new regulatory standards, review of mesh injury claims and new educational standards.
Addressing individual and community needs in the aftermath of campus sexual misconduct: restorative justice as a way forward in the reentry process^36^	2019	United States	General Academia	Theory, 1, 3	Describes the process of adapting Circles of Support and Accountability to college campuses for use in sexual misconduct cases.
Assembling Justice: Reviving Nonhuman Subjectivities to Examine Institutional Betrayal Around Sexual Misconduct^37^	2018	United States	General Academia	Legal, 2, 3	Discusses RJ as an alternative in educational environments to better tend to needs of victims.
Campus Sexual Misconduct: Restorative Justice Approaches to Enhance Compliance with Title IX Guidance^38^	2014	United States	General Academia	Theory, 2, 3	Provides in-depth argument of how RJ can become integrated into student conduct process while existing alongside current processes.
Excess Deaths From COVID-19, Community Bereavement, and Restorative Justice for Communities of Color^39^	2020	United States	Medicine	Broad	Invokes RJ principles when describing how there must be substantial healing done in regards to historical racism, both societally and in medicine.
In a Spirit of Restoration: A Phenomenology of Nursing Practice and the Criminal Justice System^40^	2018	United States	Medicine	Theory, 2	Uses RJ tenants to frame nursing within correctional facilities and the social responsibility nurses have.
Restorative Justice and Restorative History for the Sexually Transmitted Disease Inoculation Experiments in Guatemala^41^	2016	United States	Public Health	Broad	Calls for RJ in dealing with unethical inoculation experiments in Guatemala from the mid-20th century.
Restorative Justice as the Rx for Mistreatment in Academic Medicine: Applications to Consider for Learners, Faculty, and Staff^11^	2018	United States	Academic Medicine	1, 2, 3	The use of RJ to create healthier learning environments within Academic Medicine.
The devil is in the details: Exploring restorative justice as an option for campus sexual assault responses under title IX^42^	2018	United States	General Academia	Legal, 2	Discusses the benefits or RJ in addressing sexual assault on college campuses while addressing potential barriers.
The Microaggressions Triangle Model: A Humanistic Approach to Navigating Microaggressions in Health Professions Schools^43^	2020	United States	Academic Medicine	2	The use of RJ to more appropriately respond to microaggression experienced at health professional schools.
Board's eye view—Restorative justice^44^	2015	United Kingdom	Medicine	Theory	Short editorial piece advocating for use of RJ processes to reduce the inappropriate use of emergency transport services by the public.
Gross Negligence Manslaughter in Healthcare: Time for a Restorative Justice Approach?^45^	2020	United Kingdom	Medicine	Theory, Legal	Proposes RJ as a complementary process to current retributive system to deal with gross negligent manslaughter.
Human rights in childbirth, narratives and restorative justice: a review^46^	2017	United Kingdom	Public Health	Theory, 0, 1, 2	Proposes RJ processes as a way to improve the ability of legal and health care system to deal with human rights violations.
Managing complaints in health and social care^47^	2010	United Kingdom	Medicine	Theory	Discusses issues with the current system for patients to file complaints and offers RJ as a helpful perspective to improve the process.
Victims' voices, victims' interests and criminal justice in the healthcare setting^48^	2010	United Kingdom	Medicine	Legal, Theory	Advocates for RJ in health care legal practices to properly center the victim and accused to deal with their needs and perspectives.
Bringing justice to unacceptable health care services? Street-level reflections from urban South Africa^49^	2014	South Africa	Medicine	Theory	Discusses RJ's potential for healing people's relationship with a historically inequitable health system.
Mediating Conflicts in the Medical Sector: General Considerations from the Italian Perspective^50^	2015	Italy	Medicine	Theory, Legal	Discusses RJ as an alternative way to deal with medical malpractice complaints in Italy to deal with issues of defensive medicine.
Application of restorative justice values in settling medical malpractice cases^51^	2021	Indonesia	Medicine	Legal, Theory	Contextualizes RJ within the Indonesian legal system and describes its strengths when used in regards to medical malpractice cases.
“Making a place of respect”: lessons learned in carrying out consent protocol with First Nations elders^52^	2013	Canada	Research	Broad	RJ principles guiding process of making consent processes more inclusive.
Enacting research ethics in partnerships with indigenous communities in Canada: “Do it in a good way”^53^	2008	Canada	Research	Broad, 1	Utilizing RJ to facilitate discussion and active participation that creates more equitable research partnerships.
Immigrant nurses' experience of racism^54^	2001	Canada	Medicine	Broad	Peripheral mention of RJ in discussion of immigrant nurses' experiences of racism.
Restorative Justice or Restorative Health: Which Model Best Fits the Needs of Marginalized Girls in Canadian Society?^55^	2006	Canada	Public Health	Theory	Analyzes RJ as a mechanism for advocating for young women's health.
“Just culture”: Improving safety by achieving substantive, procedural and restorative justice^56^	2016	Australia	Medicine	Theory	Describes how just culture, which implements RJ principles and practices, improves workplace accountability and safety.
Justice for professional health practitioners^57^	2017	Australia	Medicine	Theory, Legal, 2	Argues that RJ is a more suitable way to deal with malpractice and other complaints against health care practitioners.
Restorative approaches to workplace bullying: educating nurses towards shared responsibility^58^	2009	Australia	Medicine	Theory, 0, 1, 2	Advocates for the use of RJ to address issues with bullying in the nursing profession.
Black Lives Matter: We are in the Same Storm but we are not in the Same Boat^59^	2020	United States	Medicine	Theory	In the context of Black Lives Matter and racial disparities, including health care, discusses how RJ can be used to deal with racial offenses.
Bringing psychological science to bear on racial health disparities: The promise of centering Black health through a critical race framework^60^	2019	United States	Medicine	Broad	While discussing implementing Critical Race Theory to psychological practice to reduce health disparities, mentions the process as a form of RJ.
Developing a Health Equity and Criminal Justice Concentration for a Master of Public Health (MPH) Program: Results from a Needs Assessment Among Community Partners and Potential Employers^61^	2019	United States	Public Health	Broad	In discussing a MPH concentration to deal with criminal justice's impact on health equity, RJ is proposed as a potential topic.
Epistemic injustice and the mental health service user^62^	2010	Ireland	Medicine	Broad	Discusses RJ in the context of patients seeking reconciliation from harm related to experiences with mental health services.
Margaret Tobin Oration abstract: Cultural changes and paradigm shifts—A challenging but rewarding journey^63^	2018	Australia	Medicine	Broad	Describes the role of RJ in supporting a Zero Suicide framework adopted by a large teaching hospital in Australia.
Nursing Leadership Implications for Clinical Placements in Corrections^64^	2020	Canada	Academic Medicine	Theory	A nursing school had clinical placements in correctional settings, and students reflected on social implications of that work, including RJ.
Changing the Culture to End Sexual Harassment: Working Group report to the Advisory Committee to the NIH Director (ACD)^28^	2019	United States	Research	Theory, 2, 3	Proposes the establishment of RJ mechanisms to properly help victims of sexual harassment within the NIH and NIH-supported institutions.
Releasing the Net to Promote Minority Faculty Success in Academic Medicine^27^	2020	United States	Academic Medicine	Broad	In discussing how to remove barriers to advancement for nonwhite faculty, mentions RJ as an important mechanism.
Restorative Just Culture: A Study of the Practical and Economic Effects of Implementing Restorative Justice in an NHS Trust^33^	2019	Australia (but actual implementation in United Kingdom)	Medicine	Theory	Restorative Just Culture was instituted at a set of health care facilities, bringing both qualitative improvements for staff and saving 1% of total costs and 2% of labor costs.
Diversity Matters: In Search of Restorative Justice^29^	2021	United States	Medicine	Theory, 2	Discusses the potential role that RJ has in dealing with racial harm that the medical system causes, specifically within the context of emergency medicine.
Inconvenient truths in suicide prevention: Why a Restorative Just Culture should be implemented alongside a Zero Suicide Framework^34^	2020	Australia	Medicine	Theory	Discusses why restorative just culture is the best option to support a zero-suicide framework model while properly supporting providers and patients.
“I Shall Be Released.” Restorative Justice Techniques Can Address Healthcare Burnout & Attrition^30^	2019	United States	Academic Medicine	Theory, 1, 2	Proposes how RJ can be used to foster connection in the health care community to help people reconnect with their purpose and reduce attrition of health care workers.
Healing the Healers^31^	2018	United States	Academic Medicine	Theory, 1, 2	Describes how RJ practices are able to be applied to deal with student and faculty misconduct within Academic Medical environments to promote a community to rebuild trust.

^a^ 1–4 Refer to the levels described in Figure 1, while “Theory” denotes an article discussing RJ theory, “Legal” indicates an article discussing RJ within legal frameworks and institutions, and “Broad” indicates the mentioning of RJ within the article.

RJ, restorative justice.

## Discussion: RJ in a Health Care Context

Most articles in this review did not discuss RJ with significant depth or describe a clear implementation. Few articles provided meaningful instruction on what RJ is. RJ, in included articles, spanned a wide range of contexts—sexual misconduct in academic environments, the health care system's interaction with communities and impact on social equity, medical school environments, malpractice and general patient complaints, human rights in health care, research ethics, and so on—indicating a breadth of application; yet, there remains a lack of a consensus on a singular definition. Confusion around what RJ is, and can be, is exemplified by Jackson and Henderson in “Restorative Justice or Restorative Health”^55^ which argues that RJ is not proactive enough for a health care application.

Their assertion suggests a misunderstanding of RJ's proactive possibilities. While the depth of discussion surrounding RJ is limited in the literature, existing work indicates the varied spaces that RJ may be applied to: educational, working environments, culture building, harm reduction, diversity and equity, health care delivery, medical and research ethics, and malpractice. Thus, while the literature is limited, it indicates that RJ is moving into academic medical spaces.

Mirroring RJ's increasing volume of literature, authors believe that RJ has substantive contributions to make to academic medicine and health care more broadly. RJ can offer a framework for responding to the damage caused by systemic racism and white supremacy culture in academic medicine, and to acknowledge ingrained processes and practices that routinely exclude marginalized populations.^65^ Similar to the Truth and Reconciliation efforts in postapartheid South Africa, the authors believe that RJ can be a powerful tool for JEDI that can positively and proactively cultivate welcoming, healthy, inclusive and affirming cultures and environments.

Provider burnout might also be improved with remedial and proactive RJ practices. This well-documented crisis^21–23^ begins as early as medical school,^66,67^ and residency.^68,69^ The crisis is complex and multifaceted, with many systemwide causes and contributors. For medical students, especially underrepresented minority and Black, Indigenous, and people of color students, mistreatment by faculty and residents correlates to shame and burnout.^70,71^ “Shame is a complex emotion in medical students that, through its destabilizing effects, can lead to withdrawal, isolation, psychological distress, altered professional identity formation, and identity dissonance.”^72^ RJ's practices and interventions could be useful strategies to build psychological safety while also creating safe spaces to be brave and authentic when navigating the toxicity of shame. For example, RJ (Tier 1) interventions have been used to address microaggressions on rounds.

In a successful RJ application, those causing the harm could glean insight about the impact of their actions in a safe environment, opening up the possibility of empathetic understanding and a change in teaching styles. More emotionally positive and empathetic environments correlate with less burnout in medical students.^71,73^ Relatedly, participatory management styles,^74^ facilitated discussions,^75^ and small group and community-building practices^76^ have been suggested as ways to improve burnout. RJ, in its proactive application, fits perfectly with these needs.

Community-Building Circles, Listening Circles, and Dialogue Circles could be used to both build community and shed light on practices and incidents that leave some of our frontline workers feeling unseen, unheard, and undervalued. If applied within the medical school and academic medical center context, not only would these educational environments be improved, but future physicians and other health care professionals would also be equipped with a powerful toolset to deal with very real, yet, often intangible issues of toxic culture in future work settings.

Barriers may remain when implementing RJ in a health care context. These include, but are not limited to, the historical hierarchies built-in and accepted by Academic Medicine and the required investment of time and resources to build reliable RJ practices. Organizations may mistakenly seek RJ as the magic bullet to address a crisis, versus investing in RJ as a mindset. Rather than viewing a 3-day RJ training or an expert RJ-interventionist as the solution, RJ must be prioritized as the compass to change organization culture and prioritize JEDI. Today, RJ Tier 0 and 1 practices present minimal investments (e.g., the integration of affective questions and statements into communication) but RJ Tier 2 practice requires more substantial investments (e.g., preparing for and hosting a restorative conference in response to malpractice).

Tier 3 offers opportunities for intentionally welcoming colleagues back to the community for example, after a long leave (e.g., family, administrative, sabbatical), or reintegrating colleagues after a disruptive event or separation (e.g., disciplinary action, internal institutional investigation, etc.) and thus proactively building a stronger community.

Another barrier would be the resistance, opposition, or subversion of the incorporation of RJ practices or a “Just Culture,” a term from safety literature that is broadly the application of RJ principles within corporate structures. One study examined a company whose management implemented Just Culture approaches, saw positive results, but prematurely interrupted progress when the values of transparency and vulnerability were required and perceived of as too high a risk, since they threatened traditional practices of being able to make decisions behind closed doors without requiring explanation.^77^ Although more outcome data are needed, evidence suggests that RJ can save health care organizations money by improving internal processes,^33^ better addressing malpractice concerns,^50,51,57^ applying effective public health interventions,^78^ improving accident processes and employee accountability,^79,80^ supporting health care workers,^34,63^ and bolstering the culture of health care.^58^

Perhaps the biggest barrier to overcome is hubris. Adopting RJ and moving toward building cultures that match the values of restorative practices and equity require leaders and institutions to explicitly acknowledge traditions that have resulted in long-standing inequities and lean into the inconvenient truths of the harms that many have experienced as a result of those traditions.

## Conclusion

RJ *is* a mindset and a practice, based on core principles, that explores harm; builds mutual understanding; invites transparency, humility, vulnerability, and accountability; and can be a powerful tool for radical authenticity, which is desperately needed for JEDI. It can be flexible enough to address specific needs of individuals, a work unit, department, academic medical centers, and health centers at micro or macro levels to influence local culture and structures. Although applications in academic medicine and health care are relatively recent, RJ has been practiced and studied longer in domains like criminal justice and K-12 education with impressive results.^78^ The recent dramatic increases in publication of RJ studies, alongside tangible programs such as the AAMC's RJAM collaborative and a series of AAMC-sponsored webinars, indicate a burgeoning desire for RJ and what it can offer within academic medical centers and health care broadly.

The “Rx for RJ” project at the University of San Diego—a multi-institutional, collaborative group—represents a formative example on tailoring RJ practices to a health care environment and is a resource for those looking to adopt RJ practices. With these resources for support, academic and health care leaders can build upon the foundation of evidence in other fields to design applications and evaluation methods specific to their health care contexts.

Although published studies on the application of RJ practices in academic medicine are somewhat limited, authors are aware that these practices are being implemented more widely than is reflected in the literature. Authors advocate for increased publication of case studies and outcomes to contribute to the literature on this topic and broader understanding of RJ's potential impact on organizational culture. Internal culture surveys, focus groups, student surveys, reports from ombudspersons, and exit interviews are all potential tools that could be leveraged to measure the impact of RJ on academic health centers. Evaluation methods should be embedded in the development of formal RJ training and implementation plans to ensure ongoing assessment of impact. In keeping with RJ's focus on community values, impact should be broadly defined to include space for individual experience and perception of organizational culture and climate.

Authors strongly believe that academic medical institutions should invest in meaningful application and evaluation of RJ because of its ability to improve overall culture for instance, through an RJ mindset (Tier 0), community-building circles (Tier 1), to expand and strengthen an institution's options when responding to harm and wrongdoings (Tier 2), reintegrating colleagues (Tier 3), and to offer the potential to proactively address pressing issues within medicine while offering health care professionals a powerful tool to carry forth into their professions.

## Ethical Approval

Not applicable.

## Supplementary Material

Supplemental data

## Supplementary Material

Supplementary Search Strategy

## Abbreviations Used

AAMC: Association of American Medical Colleges
JEDI: justice, equity, diversity, and inclusion
RJ: restorative justice
RJAM: Restorative Justice in Academic Medicine
