# Supplementary material for: Scoping Review of Restorative Justice in Academics and Medicine: A Powerful Tool for Justice Equity Diversity and Inclusion
Source: Health Equity. 2023 Sep 29;7(1):663–75. doi: 10.1089/heq.2023.0071 (PMC10541936; doi:10.1089/heq.2023.0071)
Supplement: Supplemental data [file Suppl_Data1.docx]

**Search Strategy Report:**

Topic: Scoping review of literature and current practices of Restorative Justice in Academic Medicine (RJAM)

Searcher: SJK

Date: 7.7.2021

Database (including vendor/platform): New PubMed

| Set # |  | Results |
| --- | --- | --- |
| 1 | "restorative justice"[tw] | 146 |

Database (including vendor/platform): Embase

| Set # |  | Results |
| --- | --- | --- |
| 1 | 'restorative justice'/exp OR "restorative justice":ti,ab | 162 |

Database (including vendor/platform): Scopus

| Set # |  | Results |
| --- | --- | --- |
| 1 | TITLE-ABS-KEY("restorative justice") | 2547 |
| 2 | TITLE-ABS-KEY(health OR healthcare OR medicine OR medical OR hospital OR hospitals OR doctors OR doctor OR physician OR physicians OR nurse OR nurses OR nursing) | 10054101 |
| 3 | #1 AND #2 | 167 |

Database (including vendor/platform): APA PsycInfo

| Set # |  | Results |
| --- | --- | --- |
| 1 | DE "Restorative Justice" OR (TI "restorative justice" OR AB "restorative justice") | 1111 |
| 2 | DE "Health" OR DE "Health Personnel" OR DE "Allied Health Personnel" OR DE "Caregivers" OR DE "Medical Personnel" OR DE "Mental Health Personnel" OR DE "Primary Health Care" OR DE "Health Care Services" OR DE "Behavioral Health Services" OR DE "Continuum of Care" OR DE "Electronic Health Services" OR DE "Health Care Delivery" OR DE "Hospital Programs" OR DE "Long Term Care" OR DE "Mental Health Services" OR DE "Palliative Care" OR DE "Patient Centered Care" OR DE "Prenatal Care" OR DE "Primary Health Care" OR DE "Health Care Reform" OR (TI health OR healthcare OR medicine OR medical OR hospital OR hospitals OR doctors OR doctor OR physician OR physicians OR nurse OR nurses OR nursing OR AB health OR healthcare OR medicine OR medical OR hospital OR hospitals OR doctors OR doctor OR physician OR physicians OR nurse OR nurses OR nursing) | 1,677,821 |
| 3 | S1 AND S2 | 115 |
